# Ultrasound-Guided Fine-Needle Aspiration Versus Fine-Needle Capillary Sampling in Evaluation of Lymph Node Metastasis of Thyroid Cancer

**DOI:** 10.3389/fonc.2021.642142

**Published:** 2021-04-14

**Authors:** Shujun Xia, Yilai Chen, Weiwei Zhan, Wei Zhou

**Affiliations:** ^1^ Department of Ultrasound, Rui Jin Hospital, Shanghai Jiao Tong University School of Medicine, Shanghai, China; ^2^ Department of Ultrasound, Ruijin Hospital/Lu Wan Branch, School of Medicine, Shanghai Jiaotong University, Shanghai, China

**Keywords:** efficiency, fine-needle aspiration, fine-needle capillary sampling, cervical lymph node, thyroid cancer

## Abstract

**Background:**

To compare the sampling adequacy and diagnostic efficiency of ultrasound-guided fine-needle aspiration with 22-, 25-gauge needles and capillary sampling with 22-gauge needle in the biopsy of cervical lymph node.

**Methods:**

A total of 130 cervical lymph nodes from 103 patients were consecutively included in the prospective study. Each suspected lymph node was aspirated with a 22-gauge needle, capillary sampled with a 22-gauge needle and aspirated with a 25-gauge needle. The adequacy rates and nondiagnostic rates of obtained specimen were calculated.

**Results:**

Of the 130 suspected lymph nodes, there were 77 lymph nodes<6.0 mm and 53 lymph nodes≥6.0mm in the smallest dimension. Both FNA_22G_ and FNC_22G_ got significantly higher sampling adequacy than FNA_25G_ for the total lymph nodes. For lymph nodes<6.0 mm, the sampling adequacy was significantly higher with FNA_22G_ than with FNA_25G_ for each parameter and the cumulative score (all P<0.05), while no difference were seen between FNA_22G_ and FNC_22G_, and between FNC_22G_ and FNA_25G_. There were higher nondiagnostic rates for FNA_25G_ compared with FNA_22G_ and FNC_22G_ in all lymph nodes and in each size subgroups. FNA_25G_ yielded more diagnostically inadequate specimens than FNA_22G_ and FNC_22G_ did in the total lymph nodes (P=0.002), in lymph nodes<6.0 mm (P=0.014), and in those ≥ 6.0 mm (P=0.000).

**Conclusions:**

FNA_22G_ and FNC_22G_ obtained more diagnostically adequate specimens than FNA_25G_ in cervical lymph nodes. FNA_22G_ and FNC_22G_ may be more suitable than FNA_25G_ in diagnosing cervical lymph nodes. FNA_22G_ and FNC_22G_ may yield specimens with similar quality.

## Introduction

Cervical lymph node metastasis is a common issue in papillary thyroid cancer (PTC) (1). Ultrasound (US) examination has been widely applied in evaluating the cervical lymph node metastasis caused by PTC, however, it revealed low sensitivity in these patients. US-guided fine-needle aspiration cytology (FNAC) is the first‐line diagnostic tool in the evaluation of thyroid nodules to reduce the rate of nondiagnostic and false negative results. FNA is also recommended in the evaluation of suspected lymph nodes in thyroid cancer patients (2). But the aspiration technique often leads to microscopic hemorrhages, which hinders proper cytologic interpretation. Fine-needle capillary (FNC) sampling, which is a non-aspiration technique, could reduce the amount of blood cells in specimens and produce superior quality smears according to the previous reports (3–5). However, the comparison of the efficiency between FNA and FNC in evaluating cervical lymph node has not been elucidated.

Currently, different sizes of needles are used in US-guided biopsy, ranging from 21 to 27 gauge, and different methods including aspiration and capillary sampling are applied in different occasions (6–8). Even though many advances in cancer diagnosis, there are still no uniform standard for which size and type of needle in sampling could allow for the optimal diagnosis and the least complications in evaluating cervical lymph nodes. In the current study, we aimed to compare the efficiency of US-guided FNA and FNC for cytologic diagnosis of cervical lymph node with the use of 22G and 25G needles in FNA and 22G needle in FNC. The influence of lymph node size on each method was specifically analyzed. This study would help to improve the eventual establishment of standards for needle selection on lymph node sampling.

## Method

### Patients

The study was carried out from May 2018 to Nov 2018. A total of 130 suspected cervical lymph nodes from 103 patients with PTC were consecutively enrolled in the prospective study. The lymph nodes were divided into 2 size groups based on the smallest dimension (short axis) on US: <6.0 mm and ≥6.0 mm, as the receiver operating characteristic curve (ROC) analysis showed that 6mm was the best cutoff size (9). The study was approved by the Institutional Review Board of our hospital, and all patients provided written informed consent before the sampling procedure.

### Fine-Needle Aspiration and Fine-Needle Capillary Sampling

**Figure 1:**
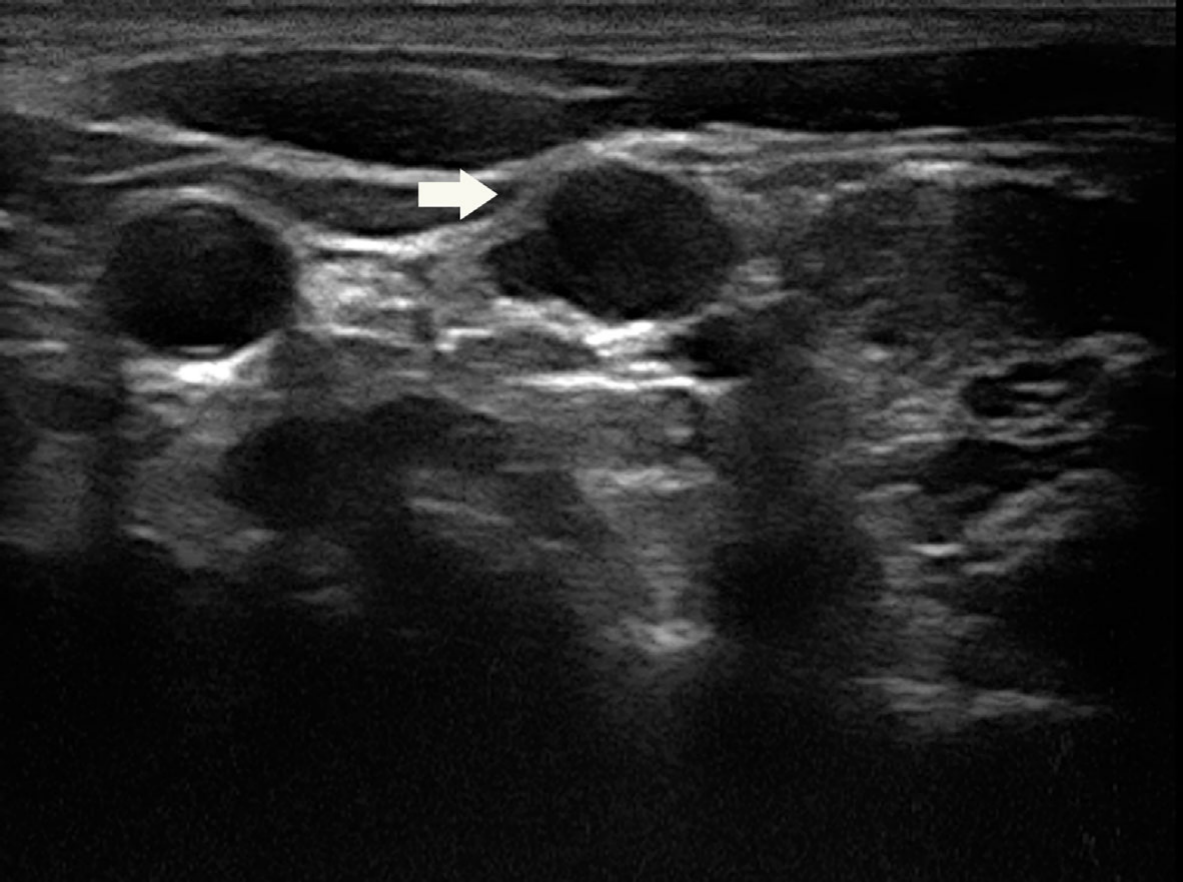
A 31-year-old woman with a suspicious lymph node (arrow) measuring 12.6×6.4×9.9mm in the left lateral compartment (level IV).

**Figure 2:**
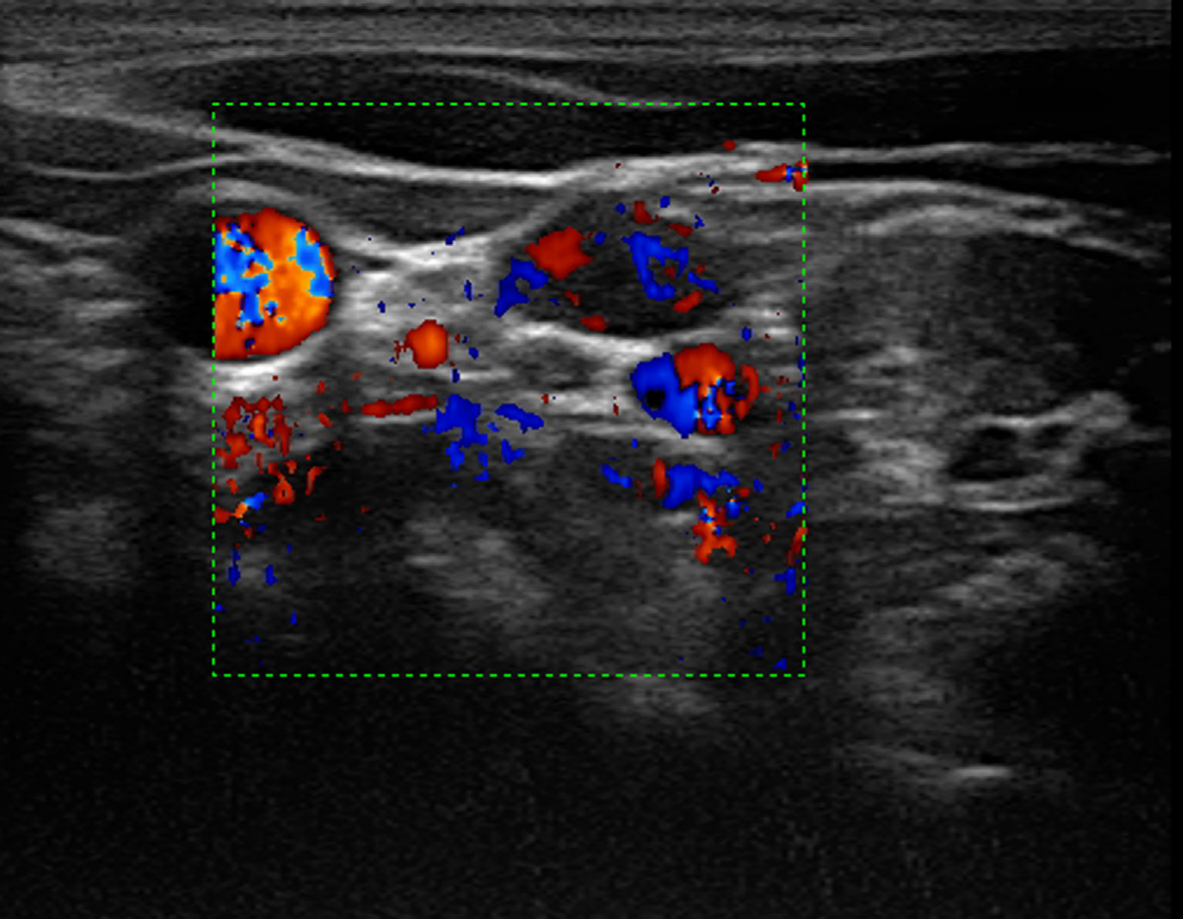
Color Doppler imaging showed rich blood supply in the lymph node. On the right side of the lymph node showed the compressed jugular vein and the carotid artery.

**Figure 3:**
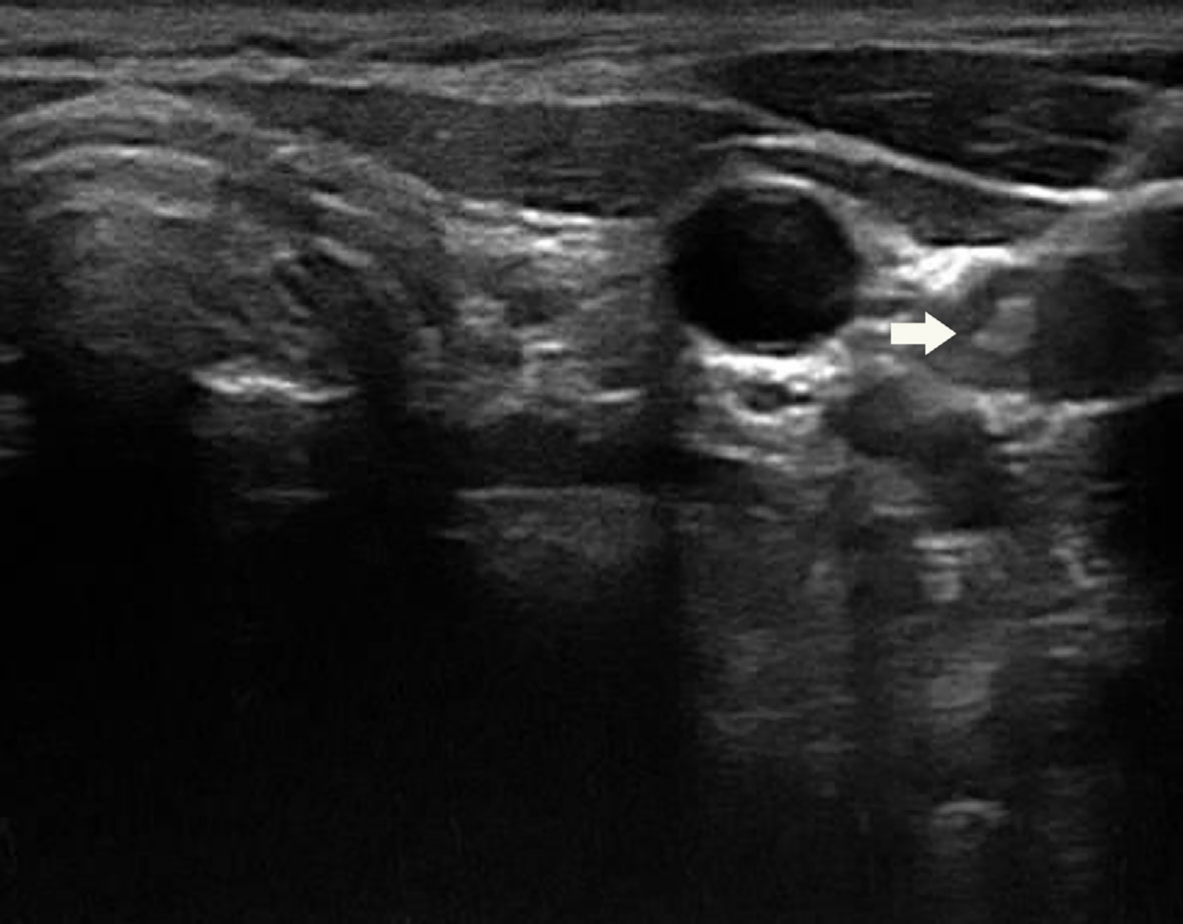
US guided fine needle aspiration biopsy was performed using a 22-gauge needle(arrow).

The procedure was conducted using Esaote MyLab 90 (Esaote, Genoa, Italy) equipped with a high‐resolution probe (LA523; 4‐13 MHz) by experienced radiologists with at least 10 years’ working in head and neck (specialized in thyroid). Each suspected lymph node was evaluated by grayscale and color Doppler US (**Figures 1** and **2**). Ultrasound features of suspicious lymph node were microcalcification, cystic aspect, peripheral vascularity, hyperechogenicity and round shape according to the American Thyroid Association (ATA) guideline (2). Then these suspicious lymph nodes were sampled under US guidance by 1 fine-needle aspiration pass with 22G needle (FNA22G) (**Figure 3**), 1 fine-needle capillary sampling with 22G needle (FNC22G) and 1 fine-needle aspiration pass with 25G needle (FNA_25G_). Among the total cases, 43 lymph nodes underwent FNA_22G,_ FNC_22G_ and FNA_25G_ sequentially; another 43 lymph nodes underwent FNC_22G,_ FNA_25G_ and FNA_22G_, sequentially; and the last 44 lymph nodes underwent FNA_25G_, FNA_22G_ and FNC_22G_ sequentially. During aspiration, the needle attached to 5‐mL syringe was inserted into the target lymph node and moved back and forth within the lymph node. Suction was applied when the needle was advanced into the lymph node and halted before the needle was removed from the lymph node. In capillary sampling, the needle, without syringe attached, was introduced into the lymph node and moved rapidly back and forth while it was angled in different directions within the lymph node. In both procedures, the needle was withdrawn when the sample material appeared in the hub of the needle.

### Interpretation of Smears

**Figure 4:**
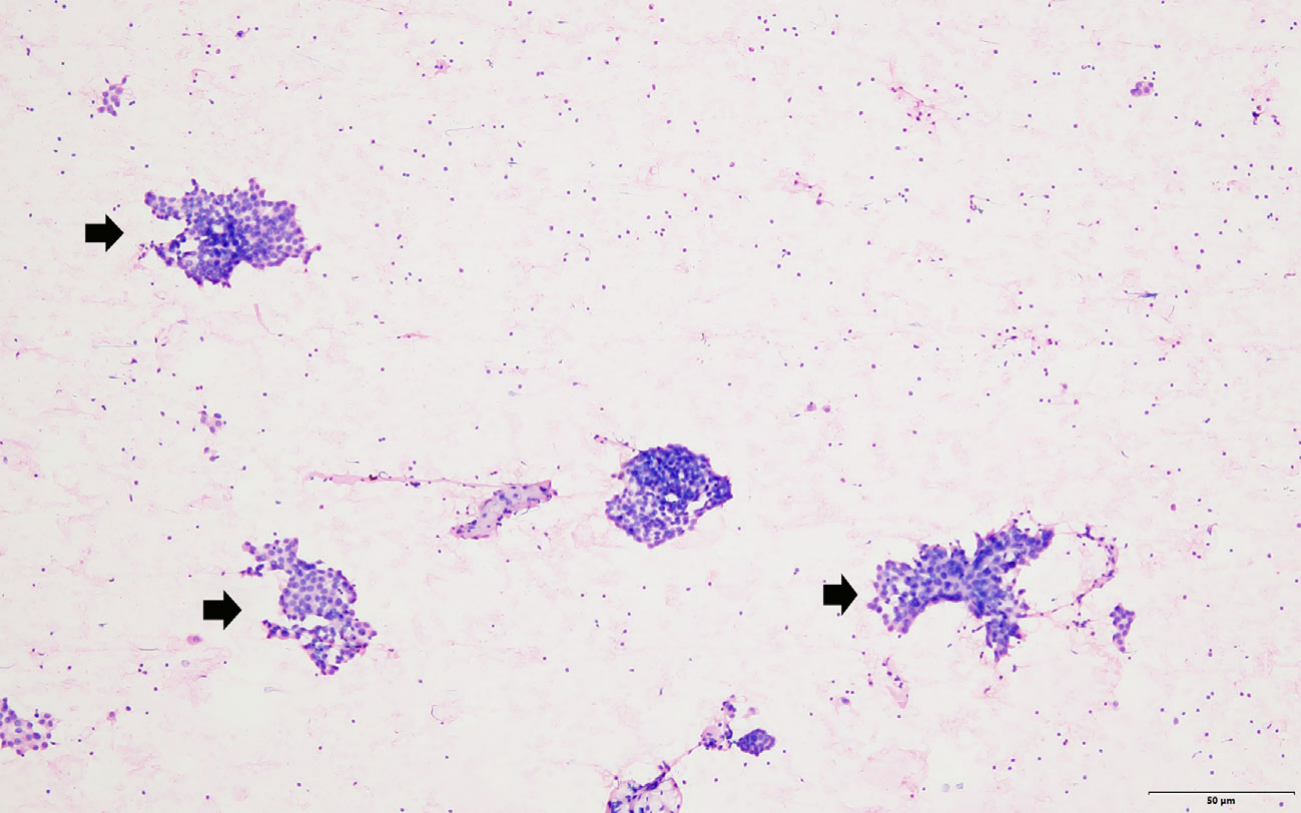
The FNA smear showed that groups of neoplastic cells (arrow head) scattered in the lymphocyte background (hematoxylin–eosin stain, magnification 100).

The obtained specimen was placed on the glass slides. Smears were prepared, fixed in alcohol, and then stained with hematoxylin and eosin. Then the slides were analyzed independently by 2 cytopathologists, and adequacy rates were calculated. The adequacy of the smears was assessed using the scoring system reported by Haddadi-Nezhad et al. (10). The adequacy of the three sampling techniques was compared using the following four parameters: background clot or blood, number of obtained cells, preserved tissue architecture, and cellular degeneration (**Table 1**). Accumulative score between 0 and 2 was categorized as “inadequate”, scores between 3 and 5 were categorized as “adequate” for diagnosis, and scores between 6 and 8 were categorized as “superior” for diagnosis (**Figure 4**).

**Table 1:** Scoring system applied to evaluate the specimen quality (10).

Parameter	Description	Scores
	Minute (easily diagnosed)	2
Background blood or clot	Intermediate (diagnosis can be made)	1
	Excessive (diagnosis cannot be made)	0
	Abundant (easily diagnosed)	2
Number of obtained cells	Intermediate (diagnosis can be made)	1
	Minimum (diagnosis cannot be made)	0
	Well preserved and easily identifiable (easily diagnosed)	2
Preserved tissue architecture	Preserved and identifiable (diagnosis can be made)	1
	Not identifiable (diagnosis cannot be made)	0
	Mild (easily diagnosed)	2
Cellular degeneration	Intermediate (diagnosis can be made)	1
	Severe (diagnosis cannot be made)	0

### Statistical Analysis

Statistical analyses were performed using the SPSS statistical software (version 24.0; SPSS Inc., Chicago, Ill). P <0 .05 was considered as statistically significant difference. The one-way ANOVA was used to compare the scores for each parameter of FNA_22G_, FNC_22G_ and FNA_25G_. The degree of agreement between the final cytopathologic diagnosis obtained by the three sampling procedures was examined using the Cohen κ statistic. The κ values were interpreted as follows: κ values from 0.00 to 0.20 indicated slight agreement; κ values from 0.21 to 0.40, fair agreement; κ values from 0.41 to 0.60, moderate agreement; κ values from 0.61 to 0.80, substantial agreement; and κ values from 0.80 to 1.00, almost perfect agreement. Chi‐square tests and Z test were applied to evaluate the differences of FNA_22G_, FNC_22G_ and FNA_25G_ regarding the nondiagnostic proportion and the overall diagnostic efficiency.

## Result

Of the 130 suspected lymph nodes from 103 patients (smallest dimension range, 2.1-28.0 mm), there were 77 lymph nodes<6.0 mm and 53 lymph nodes ≥ 6.0mm in the smallest dimension. The sampling adequacy of the 130 lymph nodes for FNA_22G_, FNC_22G_ and FNA_25G_ assessed by the four diagnostic parameters were provided in **Table 2**. The sampling adequacy from different size groups (<6.0 mm and ≥ 6.0mm) for FNA_22G_, FNC_22G_ and FNA_25G_ were showed in **Supplementary Materials S1** and **S2** respectively. For the total lymph nodes, there were no significant differences in score for each parameter or in the cumulative score between FNA_22G_ and FNC_22G_ (all P >0.05). Both FNA_22G_ and FNC_22G_ got significantly higher sampling adequacy than FNA_25G_ for the total lymph nodes. For lymph nodes that measured<6.0 mm, the sampling adequacy was significantly higher in FNA_22G_ than in FNA_25G_ for each parameter and the cumulative score (all P<0 .05), while no difference were seen between FNA_22G_ and FNC_22G_, as well as between FNC_22G_ and FNA_25G_. For lymph nodes that measured ≥6.0 mm, the sampling adequacy was not significantly different among the three procedures regarding each parameter or the cumulative score (all P > 0.05).

**Table 2:** Sampling adequacy for FNA_22G_, FNC_22G_ and FNA_25G_ assessed by each parameter in all lymph nodes.

Parameter	Score (mean ± SD)			P
	FNA_22G_	FNC_22G_	FNA_25G_	
Background blood or clot	1.415 ± 0.525	1.392 ± 0.577	1.239 ± 0.680	0.350 (FNA_22G_/FNC_22G_:0.756; FNC_22G_/FNA_25G_:0.039; FNA_22G_/FNA_25G_:0.017)
Number of obtained cells	1.423 ± 0.526	1.400 ± 0.579	1.246 ± 0.683	0.360 (FNA_22G_/FNC_22G_:0.756; FNC_22G_/FNA_25G_:0.039; FNA_22G_/FNA_25G_:0.018)
Preserved tissue architecture	1.423 ± 0.526	1.400 ± 0.579	1.246 ± 0.683	0.360 (FNA_22G_/FNC_22G_:0.756; FNC_22G_/FNA_25G_:0.039; FNA_22G_/FNA_25G_:0.018)
Cellular degeneration	1.423 ± 0.526	1.400 ± 0.579	1.246 ± 0.683	0.360 (FNA_22G/_FNC_22G_:0.756; FNC_22G_/FNA_25G_:0.039; FNA_22G_/FNA_25G_:0.018)
Cumulative score	5.685 ± 2.098	5.592 ± 2.308	4.977 ± 2.724	0.360 (FNA_22G_/FNC_22G_:0.756; FNC_22G_/FNA_25G_:0.039; FNA_22G_/FNA_25G_:0.017)

**Table 3** showed the κ scores for the agreement for total lymph nodes. The κ scores were 0.967 between FNA_22G_ and FNA_25G_, 0.897 between FNA_22G_ and FNA_25G_, 0.904 between FNA_22G_ and FNA_25G_. The agreement for cytopathological diagnosis of FNA_22G_, FNC_22G_ and FNA_25G_ in size subgroups was showed in **Supplementary Material S3**. For lymph nodes<6.0 mm, the κ scores for the agreement were 0.974, 0.923, 0.923 for FNA_22G_, FNC_22G_ and FNA_25G_ respectively. For lymph nodes≥6.0 mm, the κ scores for the agreement were 0.977, 0.921, 0.921 for FNA_22G_, FNC_22G_ and FNA_25G_ respectively.

**Table 3:** Cytopathological diagnosis of FNA_22G_, FNC_22G_ and FNA_25G_ for total lymph nodes.

Cytological diagnosis		Cases(n = 130)		κ
	FNA_22G_	FNC_22G_	FNA_25G_	
Malignant	71(54.6%)	71(54.6%)	62(47.7%)	0.967(FNA_22G_/FNC_22G_)
Benign	55(42.3%)	54(41.5%)	50(38.5%)	0.897(FNA_22G_/FNA_25G_)
Nondiagnosis	4(3.1%)^1^	5(3.8%)^2^	18(13.8%)^1,2^	0.904(FNC_22G_/FNA_25G_)

“1”significant difference between FNA_22G_ and FNA_25G_.

“2” significant difference between FNC_22G_ and FNA_25G_.

The proportion of nondiagnostic cytology was 3.1% for FNA_22G_, 3.8% for FNC_22G_, 13.8% for FNA_25G_ in all lymph nodes. The corresponding values in<6.0 mm group were 5.2%, 6.5%, 13.0%, and in≥6.0 mm group were 0%, 0%, 15.1%, respectively. A comparison indicated that the differences in the nondiagnostic proportion between FNA_22G_ and FNA_25G_, as well as between FNC_22G_ and FNA_25G_ were significantly observed. There were higher nondiagnostic rates for FNA_25G_ compared with FNA_22G_ and FNC_22G_ in all lymph nodes and in each size subgroups.

**Table 4** provided the data on the diagnostic efficiency of FNA_22G_, FNC_22G_ and FNA_25G_ among the total lymph nodes. The diagnostic efficiency of FNA_22G_, FNC_22G_ and FNA_25G_ in different size subgroups was showed in **Supplementary Material S4**. FNA_25G_ yielded significantly more diagnostically inadequate specimens than FNA_22G_ and FNC_22G_ in the total lymph nodes (P=0.002), in lymph nodes that measured<6.0 mm (P=0.014), and in lymph nodes that measured≥6.0 mm (P=0.000). However, there were no significant differences for FNA_22G_, FNC_22G_ and FNA_25G_ with regard to diagnostically superior and adequate specimens in the total lymph nodes as well as the subgroups.

**Table 4:** Comparison of Diagnostic Efficiency Between FNA_22G_, FNC_22G_ and FNA_25G_ in total lymph nodes.

Diagnostic efficiency		Total (n = 130)		P
	FNA_22G_	FNC_22G_	FNA_25G_	
Superior(6-8)	57(43.8%)	58(44.6%)	50(38.5%)	
Adequate(3-5)	71(54.6%)	66(50.8%)	62(47.7%)	
Inadequate(0-2)	2(1.5%)^a^	6(4.6%)^b^	18(13.8%)^a,b^	0.002

“a” significant difference between FNA_22G_ and FNA_25G_.

“b” significant difference between FNC_22G_ and FNA_25G_.

## Discussion

In thyroid cancer, cervical lymph node metastasis is a quite common and important issue that determines the proper surgical strategies. Ultrasound imaging, as the primary method used for the preoperative assessment of cervical lymph nodes in thyroid cancer patients, is high in specificity but low in sensitivity for evaluating lymph node metastasis (2). FNAC of the thyroid gland is an inexpensive and technically straightforward diagnostic procedure that causes little discomfort for the patient. FNA and FNC are two sampling procedures commonly used preoperatively to evaluate thyroid nodules. It was suggested that the sampling adequacy was related to the needle size, lymph node dimension, and operator’s experience etc. (11–13). The values of FNA and FNC with different needle gauges have been extensively investigated in the previous studies (14–17). However, there are relatively less studies reporting the application of FNA in suspected cervical lymph nodes even this method has been recommended in ATA guideline (2). The diagnostic performances of FNA and FNC for cervical lymph nodes have not been compared before. The current study investigated the sampling efficiency of US‐guided FNA with 22 and 25 gauge needles and FNC with 22 gauge needle in 130 cervical lymph nodes with emphasis on the influence of lymph node size, which showed that 22 gauge needle used in both FNA and FNC performed better than 25 gauge needle in FNA.

During sampling, fairly high suction pressures were often used in FNA to obtain enough materials, while FNC sampling is a non-aspiration technique (18). These two techniques have been applied in head and neck swellings and lymphadenopathies caused by a wide range of disorders (12, 19). It was reported that FNA for thyroid nodules commonly encountered the problem of blood‐stained samples, which may result in diagnostic difficulties in the cytologic interpretation. FNC relies on the property of capillary force in narrow channels, and it has been suggested that FNC may produce samples of better quality by decreasing the dilution of cells by blood (20); at the same time, there were less aspiration trauma and pain caused by FNC (4). A comparative analysis showed that FNC was technically superior with comparison to FNA in superficial lymph node lesions (5). However, the effectiveness of FNA and FNC sampling has not been fully compared in cervical lymph nodes. In the current study, FNA with 25 gauge needle yielded more inadequate samples than FNA with 22 gauge needle and FNC with 22 gauge needle. The nondiagnostic rate was higher for samples with FNA_25G_ than the other two techniques. These results were different with that of the previous studies, which indicated that FNC performed better than FNA in thyroid (7, 20). This may be because thyroid is vascular. FNA, as a suction technique, easily produces bloody specimens although it obtains more material. While obtaining more cellular architecture, more blood cells were also obtained under the suction pressure. Lymph node is an organ with less blood supply than thyroid, thus FNA for lymph node might obtain more cellular samples and better retention of architecture with less blood-stained smears (18). It was suggested that the performance of FNA and FNC varied in different organs.

The channel size of the needles is an important factor that may influence the sample quality. For FNAC of thyroid, 25–27-gauge needles were commonly used in Western countries, while 21–22-gauge needles were common in Japan (21, 22). A narrow channel(27G) was recommended for FNA of thyroid nodules in most of the studies (14, 21), because bloody smears frequently occurred using a wide channel in characteristically vascular organs like thyroid. For FNAC of suspected cervical lymph node, there was no common practice or preference of particular needle size used. In the previous studies, 18-25 gauge needles were used in some institutions (8, 9, 23). However, the optimal size of the needle for sampling lymph node has not been validated. Our study compared the needle size of FNA/FNC for cervical lymph node, showing that sampling with 22 gauge needle performed better than with 25 gauge. This may be because 22 gauge needle obtained more intact architecture and information of the samples than 25 gauge needle did. Meanwhile, there were less blood cells stained in lymph node samples using a wider channel (22 G) in FNAC.

There is a low sensitivity for ultrasound in evaluating metastatic lymph nodes of the neck (2). US-guided fine needle sampling is an efficient procedure for diagnosing suspected lymph node preoperatively. The size of the lymph node is an important factor influencing FNAC sampling. It was suggested that US-FNAC was recommended for lymph nodes ≥ 10mm (24), while some researchers suggested that patients with lymph nodes ≥ 5mm should undergo US-FNAC (25, 26). According to the ATA,ETA, and French Society of Endocrinology guidelines, suspected lymph node with the shortest dimension >5-8 mm should undergo US-FNAC (2, 27, 28). However, there is actually no agreement on the threshold value of the lymph node size. In our study, we divided the total lymph nodes into two subgroups based on 6mm, which was calculated using ROC analysis in our previous study (9). Our study indicated that the sampling adequacies were not significantly different between FNA_22G_ and FNC_22G_, while both of them were better than FNA_25G._ Thus the size may not be the major factor influencing the sampling of suspected lymph nodes.

According to 2017 American Association of Clinical Endocrinologists (AACE) guideline, suspicious lymph nodes, whatever the size, always warrant FNA according to the clinical suspicion (29). Randolph GW et al. reported that low-volume lymph node has recurrence rate (30). Alpert et al. indicated that 47% of the LNs ≤ 10mm are at risk of harboring aggressive disease biology reflected in extra-nodal extension (31). Chen YL et al. reported that the prevalence of malignant lymph nodes of thyroid cancer was higher in the small lymph nodes(<6mm) than that in the larger ones (≥6mm) when there were less than 3 suspicious US features (9). It indicated that metastasis may occur in the lymph nodes even when they were small. Therefore, it is necessary to perform sampling in all suspicious lymph node under ultrasound evaluation.

There were some limitations in this study. First, interobserver variability among radiologists who performed the sampling procedures was not assessed. Second, additional studies with larger sample sizes will be necessary to obtain more reliable results regarding the efficiency of FNA and FNC sampling in cervical lymph nodes.

## Conclusion

In conclusion, the current study demonstrated that fewer blood stains, more cells, better preserved tissue architecture, and less cellular degeneration could be obtained in FNA_22G_ and FNC_22G_ samples than in FNA_25G_ samples for the total group (all lymph nodes) and each subgroup (<6.0 mm and ≥6.0mm lymph nodes). For diagnostic efficiency, more diagnostically inadequate specimens were obtained by FNA_25G_ than by FNA_22G_ and FNC_22G_ in total group and each subgroup. Therefore, FNA with 22 gauge needle and FNC with 22 gauge needle may be more suitable than FNA with 25 gauge needle in diagnosing cervical lymph nodes. FNA and FNC with 22 gauge needle may yield specimens with similar quality.

## Data Availability Statement

The original contributions presented in the study are included in the article/**Supplementary Material**. Further inquiries can be directed to the corresponding author.

## Ethics Statement

Ethical review and approval was not required for the study on human participants in accordance with the local legislation and institutional requirements. The patients/participants provided their written informed consent to participate in this study.

## Author Contributions

WZho designed the study and reviewed the manuscript. SX and WZho did the sampling. SX wrote the manuscript. YC collected the data. WZha did the data analysis. All authors contributed to the article and approved the submitted version.

## Funding

Science Foundation for the Excellent Youth Scholars of RuiJin Hospital/Lu Wan Branch (YQA202001); Shanghai Sailing Program (19YF1431200). Shanghai Huangpu district health and family planning commission (HKM201801).

## Conflict of Interest

The authors declare that the research was conducted in the absence of any commercial or financial relationships that could be construed as a potential conflict of interest.
