# Supplementary material for: Ultrasound-Guided Fine-Needle Aspiration Versus Fine-Needle Capillary Sampling in Evaluation of Lymph Node Metastasis of Thyroid Cancer
Source: Front Oncol. 2021 Apr 14;11:642142. doi: 10.3389/fonc.2021.642142 (PMC8079778; doi:10.3389/fonc.2021.642142)
Supplement: Supplementary file 1 [file Table_1.docx]

S1 Sampling adequacy for FNA_22G_, FNC_22G_ and FNA_25G_ assessed by each parameter in lymph nodes ＜6mm

| Parameter |  | Score(mean±SD) |  | P |
| --- | --- | --- | --- | --- |
|  | FNA_22G_ | FNC_22G_ | FNA_25G_ |  |
|  |  |  |  |  |
| Background blood or clot | 1.364±0.511 | 1.351±0.580 | 1.169±0.677 | 0.077  (FNA_22G_/FNC_22G_:0.892;  FNC_22G_/FNA_25G_:0.058;  FNA_22G_/FNA_25G_:0.043) |
|  |  |  |  |  |
| Number of obtained cells | 1.377±0.514 | 1.351±0.580 | 1.182±0.683 | 0.091  (FNA_22G_/FNC_22G_:0.787;  FNC_22G_/FNA_25G_:0.080;  FNA_22G_/FNA_25G_:0.044) |
|  |  |  |  |  |
| Preserved tissue architecture | 1.377±0.514 | 1.351±0.580 | 1.182±0.683 | 0.091  (FNA_22G_/FNC_22G_:0.787;  FNC_22G_/FNA_25G_:0.080;  FNA_22G_/FNA_25G_:0.044) |
|  |  |  |  |  |
| Cellular degeneration | 1.377±0.514 | 1.351±0.580 | 1.182±0.683 | 0.091  (FNA_22G_/FNC_22G_:0.787;  FNC_22G_/FNA_25G_:0.080;  FNA_22G_/FNA_25G_:0.044) |
|  |  |  |  |  |
| Cumulative score | 5.494±2.043 | 5.403±2.318 | 4.714±2.719 | 0.086  (FNA_22G_/FNC_22G_:0.813;  FNC_22G_/FNA_25G_:0.074;  FNA_22G_/FNA_25G_:0.043) |

S2 Sampling adequacy for FNA_22G_, FNC_22G_ and FNA_25G_ assessed by each parameter in lymph nodes ≥6mm

| Parameter |  | Score(mean±SD) |  | P |
| --- | --- | --- | --- | --- |
|  | FNA_22G_ | FNC_22G_ | FNA_25G_ |  |
|  |  |  |  |  |
| Background blood or clot | 1.491±0.541 | 1.453±0.574 | 1.340±0.678 | 0.406  (FNA_22G_/FNC_22G_:0.747;  FNC_22G_/FNA_25G_:0.333;  FNA_22G_/FNA_25G_:0.198) |
|  |  |  |  |  |
| Number of obtained cells | 1.491±0.541 | 1.472±0.575 | 1.340±0.678 | 0.373  (FNA_22G_/FNC_22G_:0.872;  FNC_22G_/FNA_25G_:0.260;  FNA_22G_/FNA_25G_:0.198) |
|  |  |  |  |  |
| Preserved tissue architecture | 1.491±0.541 | 1.472±0.575 | 1.340±0.678 | 0.373  (FNA_22G_/FNC_22G_:0.872;  FNC_22G_/FNA_25G_:0.260;  FNA_22G_/FNA_25G_:0.198) |
|  |  |  |  |  |
| Cellular degeneration | 1.491±0.541 | 1.472±0.575 | 1.340±0.678 | 0.373  (FNA_22G_/FNC_22G_:0.872;  FNC_22G_/FNA_25G_:0.260;  FNA_22G_/FNA_25G_:0.198) |
|  |  |  |  |  |
| Cumulative score | 5.962±2.166 | 5.868±2.287 | 5.359±2.711 | 0.381  (FNA_22G_/FNC_22G_:0.840;  FNC_22G_/FNA_25G_:0.276;  FNA_22G_/FNA_25G_:0.197) |

S3 Cytopathological diagnosis of FNA_22G_, FNC_22G_ and FNA_25G_ for lymph nodes based on size subgroups

| Cytological diagnosis | Cases（n=77）  ＜6mm | | | κ | Cases（n=53）  ≥6mm | | | κ |
| --- | --- | --- | --- | --- | --- | --- | --- | --- |
|  | FNA_22G_ | FNC_22G_ | FNA_25G_ |  | FNA_22G_ | FNC_22G_ | FNA_25G_ |  |
| Malignant | 42(54.5%) | 41(53.2%) | 37(48.0%) | 0.974  (FNA_22G_/FNC_22G_) | 29(54.7%) | 30(56.6%) | 25(47.2%) | 0.977  (FNA_22G_/FNC_22G_) |
| Benign | 31(40.3%) | 31(40.3%) | 30(39.0%) | 0.923  (FNA_22G_/FNA_25G_) | 24(45.3%) | 23(43.4%) | 20(37.7%) | 0.921  (FNA_22G_/FNA_25G_) |
| Nondiagnosis | 4(5.2%)^3^ | 5(6.5%)^4^ | 10(13.0%)^3,4^ | 0.932  (FNC_22G_/FNA_25G_) | 0(0.0%)^5^ | 0(0.0%)^6^ | 8(15.1%)^5,6^ | 0.921  (FNC_22G_/FNA_25G_) |

“3” significant difference between FNA_22G_ and FNA_25G_ in＜6mm group

“4” significant difference between FNC_22G_ and FNA_25G_ in＜6mm group

“5” significant difference between FNA_22G_ and FNA_25G_ in ≥ 6mm group

“6” significant difference between FNC_22G_ and FNA_25G_ in ≥ 6mm group

S4 Comparison of Diagnostic Efficiency Between FNA_22G_, FNC_22G_ and FNA_25G_ based on size subgroups

| Diagnostic efficiency |  | Cases（n=77）  ＜6mm |  | P |  | Cases（n=53）  ≥6mm |  | P |
| --- | --- | --- | --- | --- | --- | --- | --- | --- |
|  | FNA_22G_ | FNC_22G_ | FNA_25G_ |  | FNA_22G_ | FNC_22G_ | FNA_25G_ |  |
| Superior  （6-8） | 30(39.0%) | 31(40.3%) | 26(33.8%) |  | 27(54.5%) | 27(53.2%) | 24(48.1%) |  |
|  |  |  |  |  |  |  |  |  |
| Adequate（3-5） | 46(59.7%) | 42(54.5%) | 39(50.6%) |  | 25(40.3%) | 24(40.3%) | 23(39.0%) |  |
|  |  |  |  |  |  |  |  |  |
| Inadequate（0-2） | 1(1.3%)^c^ | 4(5.2%)^d^ | 12(15.6%)^c,d^ | 0.014 | 1(5.2%)^e^ | 2(6.5%)^f^ | 6(13.0%)^e,f^ | 0.000 |

“c” significant difference between FNA_22G_ and FNA_25G_ in＜6mm group

“d” significant difference between FNC_22G_ and FNA_25G_ in＜6mm group

“e” significant difference between FNA_22G_ and FNA_25G_ in ≥ 6mm group

“f” significant difference between FNC_22G_ and FNA_25G_ in ≥ 6mm group
